# Cytopathic effect of *Acanthamoeba* on human corneal fibroblasts

**Published:** 2012-08-09

**Authors:** Noriko Takaoka-Sugihara, Satoru Yamagami, Seiichi Yokoo, Masao Matsubara, Kenji Yagita

**Affiliations:** 1 Department of Ophthalmology, Tokyo Women's Medical University Medical Center East, Tokyo Japan; 2 Department of Parasitology, National Institute of Infectious Diseases, Tokyo, Japan; 3 Corneal Regeneration Research Team, Foundation for Biomedical Research and Innovation, Kobe Japan; 4 Department of Ophthalmology, Tokyo University Graduate School of Medicine, Tokyo, Japan

## Abstract

**Purpose:**

*Acanthamoeba* keratitis is associated with keratocyte depletion in humans. We investigated how *Acanthamoebae* isolated from corneas affected by *Acanthamoeba* keratitis interacted with human corneal stromal cells in vitro.

**Methods:**

*Acanthamoebae* were isolated from 6 patients with *Acanthamoeba* keratitis and genotyping was done. Whether the isolated *Acanthamoebae* could invade the corneal stroma was assessed with denuded corneal stroma ex vivo. The cytopathic effect of *Acanthamoeba* on cultured corneal fibroblasts from donor corneas was quantitatively evaluated by the MTT assay after culture under various conditions. Terminal deoxynucleotidyl transferase-mediated dUTP nick-end labeling (TUNEL) and Annexin V staining were employed to detect apoptotic cells among the corneal fibroblasts co-cultured with *Acanthamoebae*.

**Results:**

All 6 *Acanthamoebae* isolated from the patients with *Acanthamoeba* keratitis were shown to have the T4 genotype by 18S rDNA sequence analysis. *Acanthamoebae* invaded the denuded corneal stroma in the ex vivo experiments and had a cytopathic effect on human corneal fibroblasts after direct adhesion, but not via chemical mediators. A cytopathic effect was detected with all 6 *Acanthamoebae* and corneal fibroblasts mainly died by apoptosis, as evidenced by Annexin V staining.

**Conclusions:**

*Acanthamoebae* isolated from patients with *Acanthamoeba* keratitis had a cytopathic effect on human corneal fibroblasts, mainly via induction of apoptosis after direct adhesion. Our findings may provide some clues to the pathophysiology of corneal keratocyte depletion in patients with *Acanthamoeba* keratitis.

## Introduction

*Acanthamoebae* are free-living cyst-forming protozoans that can cause painful keratitis with the potential loss of vision [1,2]. *Acanthamoeba* keratitis (AK) is closely associated with use of contact lenses, but can also occur in non-contact lens wearers after corneal trauma or exposure to contaminated water [3-5]. *Acanthamoebae* are classified into 15 genotypes (T1-T15), among which the T4 genotype has been identified as the major cause of AK [6,7].

The human corneal epithelium contains immunoprotective dendritic cells as the first line of defense against corneal infection [8]. Creation of corneal epithelial damage before the application of *Acanthamoeba*-infected contact lenses is essential for the development of AK in experimental models [9,10]. Because AK is rare in comparison with the very large number of contact lens wearers, the occurrence of corneal epithelial damage seems to be a precondition leading to AK. Therefore, the interaction between *Acanthamoeba* and keratocytes could provide clues to the mechanism underlying the development of AK. *Acanthamoeba* has a cytopathic effect on various cells [11-16], and keratocyte depletion by invading trophozoites has been detected by histological examination of human corneas with AK [17], but it is still unknown whether *Acanthamoeba* has a direct or indirect effect on human corneal fibroblasts.

In the present study, we isolated *Acanthamoebae* from the corneas of patients with AK and obtained activated keratocytes (i.e., corneal fibroblasts) from human donor corneas. Then we investigated whether *Acanthamoeba* had any effect on cultured corneal fibroblasts. Our findings suggested that *Acanthamoeba* has a direct adverse influence on the survival of corneal fibroblasts.

## Methods

### Isolation of *Acanthamoebae*

*Acanthamoeba* isolates were obtained from the corneal scrapings of 6 patients with AK at the Medical Center East of Tokyo Women’s Medical University. The isolates were grown on non-nutrient agar plates with heat-killed *Escherichea coli* as a source of nutrients and were designated as follows: E44, E46, E51, E52, E57, and E58. This research was done according to the tenets of the Declaration of Helsinki and was approved by the institutional review board of Tokyo Women’s Medical University. Written informed consent was obtained from the patients with AK.

### Isolation and culture of human corneal fibroblasts

Human corneas for research were obtained from the Northwest Lions Eye Bank (Seattle, WA) and the tissue outside the sclerocorneal button was removed. The endothelial layer of the cornea with Descemet’s membrane was removed as a sheet, and then the corneal epithelium was removed mechanically. Denuded corneas were treated with collagenase (2 mg/ml in low glucose D-MEM; Wako, Osaka, Japan) at 37 °C until a single cell suspension of corneal keratocytes was obtained. Then human corneal fibroblasts were cultured from the corneal keratocytes in D-MEM with 10% fetal bovine serum (FBS) at 37 °C under 5% CO_2_ in 6-cm diameter dishes (Thermo Fisher Scientific, Roskilde, Denmark), glass-bottomed culture dishes (MatTek Corporation, Ashland, MA), and 24-well plates (Thermo Fisher Scientific). Cells were used for the present experiments after two to four passages.

### Isolation and sequencing of *Acanthamoeba* DNA

*Acanthamoebae* were grown in PYGC medium (10 g proteose peptone, 10 g yeast extract, 1 g glucose, 5 g NaCl and 1 g L-Cysteine in 1,000 ml of 5 mM phosphate buffer pH 7.0) [18] at 30 °C for 3–4 days in 25 cm^2^ culture flasks, and then were harvested and washed with phosphate-buffered saline (PBS). DNA was extracted using the QIAmp DNA mini kit^®^ (Qiagen, Valencia, CA) according to the manufacturer’s instructions. To identify each *Acanthamoeba* strain, a fragment of the 18S rDNA gene was amplified using two *Acanthamoeba*-specific primers (JDP1 and JDP2) [19,20]. A 25 µl reaction mixture including 1 µl of extracted DNA was prepared and PCR was performed with a thermal cycler (GeneAmp PCR System; Applied Biosystems, Carlsbad, CA) using 45 cycles of 94 °C for 30 s, 60 °C for 45 s, and 72 °C for 30 s.

The amplified fragments of 18S rDNA were visualized by 1.5% agarose gel electrophoresis with ethidium bromide staining and compared to DNA size markers. Amplicons were analyzed with a 310 ABI PRISM automated sequencer (Applied Biosystems) and the sequences obtained were compared with those published in sequence databases (e.g., Genebank) by using the BLAST search program. The genotype of each isolate was identified by comparison with the previously reported reference sequences of each T type.

### Ex vivo invasion of *Acanthamoeba* into corneal stroma

Denuded corneas without epithelial or endothelial cells were placed endothelial side up in the wells of a 24-well plate containing D-MEM. *Acanthamoebae* (1×10^5^; >95% trophozoites) were added to the center of each cornea and incubated at 37 °C under serum-free conditions. Two days later, the corneas were fixed in 10% formalin and stained with hematoxylin and eosin (HE) solution for light microscopy. Besides, *Acanthamoebae* were checked not to transform into cyst form in 37 °C condition.

### Cytopathic effect of *Acanthamoeba* on corneal fibroblasts

After human corneal fibroblasts were grown to form confluent monolayers, the culture dishes were washed three times with PBS to remove the medium containing FBS and then serum-free D-MEM was added. Next, *Acanthamoebae* (1×10^3^ E44, >95% trophozoites) were carefully added to the center of each 6-cm diameter dish and incubated at 37 °C for 2 days.

To test whether the cytopathic effect of *Acanthamoeba* was due to direct adherence to corneal fibroblasts or to soluble factors such as chemical mediators, we used insert culture dishes with 0.4 µm pores (Transwell; Corning Life Sciences, Corning, NY), and corneal fibroblasts were cultured in the outer plate. *Acanthamoebae* could not pass through these pores under any culture conditions (data not shown). We used 6-well plates with insert culture dishes to investigate the morphological change of corneal fibroblasts. We prepared four times as many *Acanthamoebae* (4×10^4^) as 24-well plates (1×10^4^) based on our experiments, because the well size of 6-well plate is about four times as much as one of 24-well plate. The number of 1×10^4^ *Acanthamoeba* used in 24-well plate was the highest number used in our experiments. Corneal fibroblasts were harvested by using 0.05% trypsin. The optical density of each well was read using a Cell Proliferation Kit I (MTT assay; Roche Molecular Biochemicals, Mannheim, Germany) at 655 nm in a microplate reader (Bio-Rad Laboratories, Hercules, CA) to quantify viable cells.

Next, *Acanthamoebae* (1.3×10^3^ to 1×10^4^) were added to 4 wells of a 24-well plate and incubated at 37 °C for 2 days. Dishes without *Acanthamoebae* were used as the negative control. After incubation, corneal fibroblasts were harvested by using 0.05% trypsin and the optical density was determined with the 3-[4,5-dimethylthiazol-2-yl]-2,5-diphenyl tetrazolium bromide (MTT) assay. This assay was done in the 6 different isolates of *Acanthamoeba* using quadricate samples (1×10^4^ of E44, E46, E51, E52, E57, and E58).

### TUNEL assay

Corneal fibroblasts were cultured in 5 ml flasks (Thermo Fisher Scientific) until confluence and 1×10^5^ *Acanthamoebae* (E44) were added. After 4 days, cells were collected and the terminal deoxynucleotidyl transferase (TdT)-mediated dUTP nick-end labeling (TUNEL) assay was done to detect DNA fragmentation in apoptotic cells using an in situ Apoptosis Detection Kit (TaKaRa, Shiga, Japan). Corneal fibroblasts cultured without *Acanthamoebae* were used as the negative control, while corneal fibroblasts in dishes with Actinomysin D, *Streptomyces* sp. (MERCK, Darmstadt, Germany) were the positive control.

### Quantification of apoptotic cells

*Acanthamoebae* (1×10^4^) were added to human corneal fibroblasts and cultured at 37 °C for up to 5 days under serum-free conditions. On days 0, 1, 2, 3, 4, and 5 of incubation, the corneal fibroblasts were harvested using 0.05% trypsin-EDTA (Invitrogen, Tokyo, Japan) and were centrifuged at 200× g for 5 min at 4 °C. After removal of the supernatant, cells were labeled with Annexin V-FITC and propidium iodide (PI) by using an Annexin V-FITC apoptosis detection kit (Beckman Coulter, Brea, CA) according to the manufacturer’s instructions. Fluorescence from FITC and PI was detected under a fluorescence microscope (Axioplan 2 Imaging; Micro-optik, Deursen, Netherlands) at 518 nm and 620 nm, respectively. FITC/Annexin V-positive cells were counted in ten fields because these cells were regarded as being in the initial stage of apoptosis. FITC/Annexin V-negative and PI-negative (unstained) cells were defined as viable cells. While FITC/Annexin V-positive cells showing green fluorescence were regarded as early apoptotic cells, FITC/Annexin-positive and PI-positive cells showing red and green fluorescence were regarded as necrotic cells, because PI-positive cells include both necrotic cells and cells that have gone through apoptosis. The percentage of apoptotic cells among total cells was calculated.

### Statistical analysis

Statistical comparisons between two groups were performed by the Mann–Whitney U-test. For multiple comparisons among groups, one-way ANOVA was used and then a post-hoc least significant difference test was performed. Statistical significance was set at p<0.05.

## Results

### Genotyping of *Acanthamoebae* isolates

The isolates were termed E44, E46, E51, E52, E57, and E58. Based on 18S rDNA sequence analysis, all of these *Acanthamoebae* isolates belonged to the T4 genotype, but their sequences varied. Table 1 summarizes the results of genotyping and the BLAST search findings. The sequences of the isolates showed 98~100% correspondence with those of clinical isolates reported previously.

**Table 1:** Results of genotyping of the isolates and BLAST search.

**Isolate**	**Sex**	**Age (y)**	**Strain [GeneBank]**	**Tissue source**	**Homology**	**Genotype**
E44	F	35	ATCC 50497 [U07410]	cornea	100%	T4
E46	M	28	ATCC 30461 [AY026243]	cornea	99%	T4
E51	F	34	AC 29 [AB554228]	cornea	99%	T4
E52	M	59	CDC V390 [AY703004]	cornea	99%	T4
E57	F	47	CDC V062 [AY702989]	cornea	100%	T4
E58	M	17	CDC V029 [U07402]	cornea	98%	T4

We summarized the results of each isolates. E44, 46, 51, 52, 57, and 58. Those isolates were analyzed based on 18S rDNA and compared with isolates reported previously. “Strain” shows isolates that had high homology(“Homology”) with isolates examined in our hospital. “Tissue source ” shows the organ where “Strain ” isolates were obtained. “Genotype” shows each isolates’ genotype in 18S rDNA classification. All isolates were T4 genotype. F; female, M; male, CDC; Centers for Disease Control and preventation, ATCC; American Type Culture Collection.

### Effect of *Acanthamoeba* on denuded human corneal stroma

**Figure 1:**
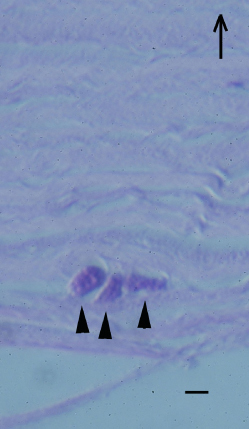
Ex vivo invasion of *Acanthamoeba* into corneal stroma. *Acanthamoebae* were added to denuded human corneal stroma. *Acanthamoebae* were placed on the denuded corneal stroma (endothelial side up) and incubated at 25 °C for 2 days. Hematoxylin and eosin staining shows *Acanthamoebae* (arrowheads) located in fine collagen fibrils. Arrow shows the direction of corneal epithelium. Scale bar=10 µm.

We used the E44 isolate throughout our experiments because it was the first *Acanthamoeba* isolated at our hospital that had the typical morphological and proliferative features of *Acanthamoebae*. To test whether E44 could invade human corneal stroma, trophozoites were added to denuded corneal stroma and incubated at 37 °C for 2 days. HE staining showed *Acanthamoebae* in the inside the corneal stroma (Figure 1), indicating that the E44 isolate of *Acanthamoeba* could attach to the corneal surface and invade the corneal stroma through fine collagen fibrils. Similar findings were detected in different three donor corneas.

### Cytopathic effect on corneal fibroblasts

**Figure 2:**
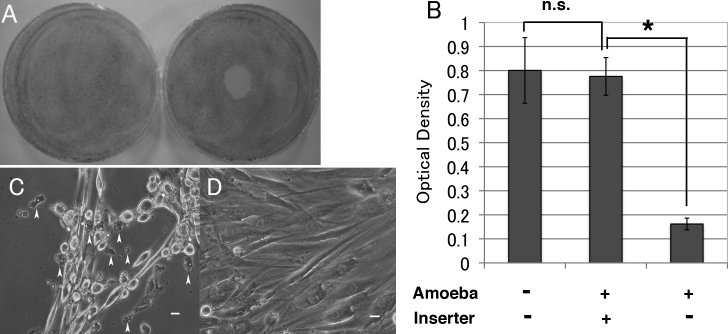
Direct and indirect cytopathic effects of *Acanthamoeba* on corneal fibroblasts. **A**: *Acanthamoebae* were placed on corneal fibroblasts at the center of 6-cm dishes and incubated at 25 °C for 2 days. The fibroblasts are uniformly stained with Giemsa solution in a control dish (left). The central area where *Acanthamoebae* were placed shows no staining (indicating loss of corneal fibroblasts) in a treated dish (right). **B**: MTT assay showed there was no significant difference of optical density value in the outer dishes with corneal fibroblasts with or without insert culture dishes bearing *Acanthamoebae*. Significant low optical density value is detected in *Acanthamoebae* direct adhesion group, compared with insert culture dishes bearing *Acanthamoebae.* (n=6) Amoeba; Acanthamoeba, Inserter; insert culture dish. **C**: Phase contrast microscopy shows many corneal fibroblasts are detached and *Acanthamoebae* adhere to corneal fibroblasts and the dish surface. Arrowheads show active *Acanthamoebae* co-cultured with corneal fibroblasts. **D**: Confluent human corneal fibroblasts are seen. *Acanthamoebae* in the insert culture dishes with 0.4 µm pores are not observed. Similar findings were obtained with repeated two sets of experiments. Representative data are shown. Scale bar=10 µm.

To investigate the effect of *Acanthamoeba* on corneal fibroblasts, isolates were added to the center of 6-cm dishes containing human corneal fibroblasts and were stained with Giemsa solution after culture. In this experiment, *Acanthamoebae* were placed carefully on the center of the dish not to spread to the periphery. It was found that fibroblasts had disappeared from the center of the dishes and this central area with no staining (Figure 2A, right) corresponded to the site where *Acanthamoebae* were placed. Although corneal fibroblasts on the peripheral area should have been exposed to soluble factors *Acanthamoebae* produced, there was no apparent change in the outside area of corneal fibroblasts at least 2 days after *Acanthamoebae* addition, suggesting that direct adhesion of *Acanthamoebae* to corneal fibroblasts is essential for the cytopathic effect of *Acanthamoebae* and soluble factors *Acanthamoebae* produced does not affect the fate of corneal fibroblasts.

To test whether the cytopathic effect of *Acanthamoeba* was due to direct adherence to corneal fibroblasts or to soluble factors such as chemical mediators, we used insert culture dishes with 0.4 µm pores, and corneal fibroblasts were cultured in the outer plate. *Acanthamoebae* could not pass through these pores under any culture conditions. MTT assay showed there was no significant difference of optical density value in the outer dishes with corneal fibroblasts with or without insert culture dishes bearing *Acanthamoebae*. However, significant low optical density value was detected in *Acanthamoebae* direct adhesion group, compared with insert culture dishes bearing *Acanthamoebae* (Figure 2B). When *Acanthamoebae* were placed directly on the fibroblasts in the well without insert culture dishes, several fibroblasts were detached from dishes (Figure 2C). In contrast, corneal fibroblasts adhered surface of dishes and cell viability was maintained up to 7 days (Figure 2D) in case that insert culture dishes containing *Acanthamoebae* were placed in the wells, indicating that direct contact with *Acanthamoebae*, but not soluble factors, was essential for the cytopathic effect on corneal fibroblasts.

**Figure 3:**
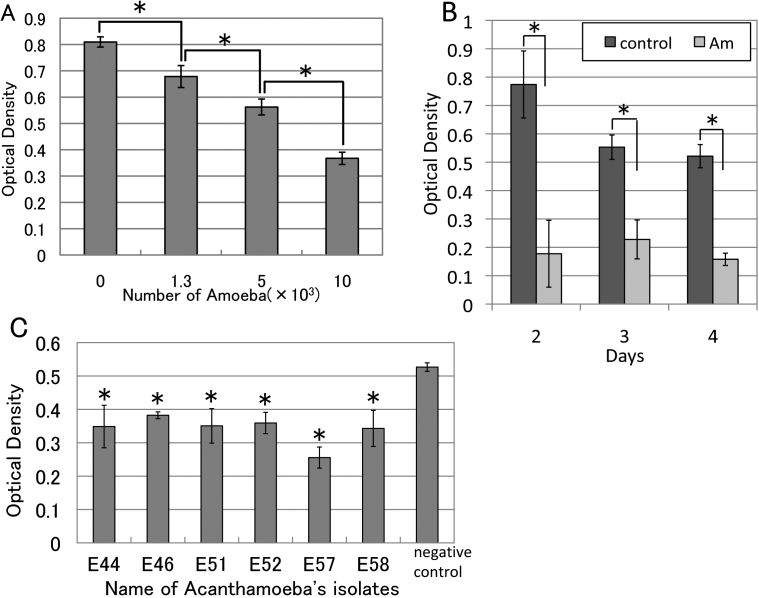
Cytopathic effect of *Acanthamoeba* on corneal fibroblasts in various conditions. **A**: *Acanthamoebae* (0 to 10×10^3^) were added to corneal fibroblasts in each well and incubated at 25 °C for 2 days. *Acanthamoebae* (1×10^4^) significantly decreased the viability of corneal fibroblasts compared with no *Acanthamoebae* (n=4). **B**: A significant decrease of corneal fibroblast viability was detected from day 2 (n=4). **C**: Cytopathic effect on corneal fibroblasts for 6 *Acanthamoebae* isolates from our AK patients. A significant decrease of optical density (indicating a cytopathic effect on corneal fibroblasts) was detected with all tested *Acanthamoebae* compared to control cultures with no *Acanthamoebae* (n=4). Similar findings were obtained with repeated two experiments. Representative data are shown. *p<0.05.

We also investigated whether an increase in the number of *Acanthamoebae* could affect the viability of corneal fibroblasts. We found that viable corneal fibroblasts decreased significantly in a dose-dependent manner after co-culture with *Acanthamoebae* (Figure 3A). We analyzed corneal fibroblast viability over time using 1×10^4^ *Acanthamoebae*, because larger numbers of *Acanthamoebae* were too toxic for the fibroblasts. As shown in Figure 3B, the viability of corneal fibroblasts cultured with *Acanthamoebae* was significantly decreased from day 2 compared with the control cultures. Next, we evaluated the cytopathic effect of various T4 genotypes isolated from patients with AK. Significant decreases of corneal fibroblast viability were detected with all *Acanthamoeba* strains tested as compared to culture without *Acanthamoebae* (Figure 3C), suggesting that *Acanthamoeba* isolates from AK patients have a similar cytopathic effect on human corneal fibroblasts.

### Detection of apoptotic corneal fibroblasts

**Figure 4:**
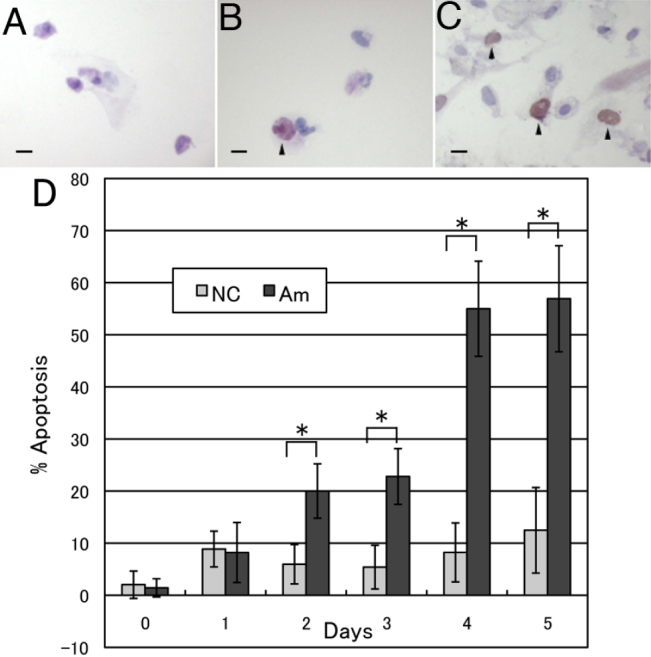
Detection of apoptotic human corneal fibroblasts. TUNEL staining was performed after human corneal fibroblasts were co-cultured with *Acanthamoebae*. TUNEL-positive cells are not detected in cultures of fibroblasts without *Acanthamoebae* (**A**). Arrowheads show TUNEL-positive apoptotic corneal fibroblasts cultured with *Acanthamoebae* (**B**) and with Actinomysin D (**C**). Scale bar=100 µm. **D**: Percentage of Annexin V-positive cells. A significant increase of Annexin V-positive corneal fibroblasts was detected on day 2 or later of culture with *Acanthamoebae*. More than 50% of corneal fibroblasts were Annexin V-positive on days 4 and 5 (n=4–5). Similar results were obtained with repeated two experiments. NC; Negative control, corneal fibroblasts without *Acanthamoebae*, Am; Corneal fibroblasts co-cultured with *Acanthamoebae*. *p<0.05.

To test whether or not apoptosis of corneal fibroblasts was induced by *Acanthamoeba* infection, co-culture of *Acanthamoeba* with human corneal fibroblasts was done and TUNEL staining was performed to detect DNA fragmentation. As a result, DAB-positive apoptotic corneal fibroblasts were found in the positive control cultures and the cultures with *Acanthamoeba*, but not in the negative control cultures (Figure 4A-C). Next, to evaluate the extent of apoptosis when corneal fibroblasts were cultured with *Acanthamoebae*, we calculated the percentage of cells stained with Annexin V, which identifies early apoptosis (Figure 4D). Annexin V-positive cells increased significantly compared with necrotic cells on days 2 to 5. More than 50% of cells were Annexin V-positive on days 4 and 5, indicating that *Acanthamoeba* mainly killed corneal fibroblasts by apoptosis.

## Discussion

All 6 *Acanthamoeba* isolates from the corneas of our AK patients had the T4 genotype according to 18S rDNA sequence analysis, as shown in Table 1. Our present findings clearly demonstrated that all of the *Acanthamoeba* isolated from AK patients had a cytopathic effect on human corneal fibroblasts and that this cytopathic effect was due to direct adhesion to corneal fibroblasts rather than soluble factors, suggesting that the keratocyte depletion demonstrated by histological examination of human corneas with AK [17] is induced by direct adhesion of *Acanthamoeba* to activated corneal keratocytes (i.e., corneal fibroblasts). DNA fragmentation was detected in corneal fibroblasts cultured with *Acanthamoebae* and the fibroblasts were mainly depleted by apoptosis, as evidenced by Annexin V staining to detect early apoptosis. In vitro observation showed active *Acanthamoeba* trophozoites phagocytosing fragments of corneal fibroblasts (unpublished observation 2010). These findings may reproduce those occurring in AK induced by pathogenic *Acanthamoeba*.

Our in vitro study suggested that activated corneal fibroblasts may be extremely vulnerable to *Acanthamoeba* infection in vivo. However, AK is localized to the central area of the cornea and does not expand to the periphery or the conjunctiva. This may be because all layers of the peripheral corneal stroma contain monocytes [21] and the substantia propria of the conjunctiva has several leukocytes (mainly macrophages) [19]. Moreover, corneal fibroblasts have the potential to produce abundant chemokines that attract macrophages and neutrophils [21-23]. Clinically, vascular invasion of AK lesions dramatically suppresses disease activity, implying a critical role of leukocytes from the blood vessels in combating AK. Thus, differences in the immunoprotective microenvironment of the ocular surface may confine AK lesions to the central area of the avascular cornea, but severe visual impairment due to corneal scarring is a common problem. These findings suggest that local application of host leukocytes and use of chemokines to attract neutrophils and macrophages may be treatment options for AK that is uncontrolled by current therapies.

Sequencing of nuclear 18S rDNA [6,7] is a useful method of classifying *Acanthamoebae* accurately. Fifteen types of *Acanthamoebae* (T1 to T15) have been identified, but isolates obtained from AK are mainly of the T4 genotype. All 6 isolates obtained at our hospital were of the T4 genotype and all isolates had a cytopathic effect on corneal fibroblasts (Figure 3C). However, the extent of the cytopathic effect varied, suggesting pathophysiological diversity of *Acanthamoebae* with the T4 genotype. Therefore, we retrospectively examined the correlation between the cytopathic effect in vitro and clinical severity of AK caused by each isolate, but no correlation was detected in our series (unpublished observation). This may be because clinical severity of AK depends on various factors, such as the stage at the first visit to hospital, previous treatment, and use of topical corticosteroids due to misdiagnosis. However, detailed classification of *Acanthamoeba* by DNA typing may contribute to prediction of the prognosis and selection of appropriate treatment for each isolate in the near future.

The following limitations of our study should be noted. Cultured human corneal fibroblasts are not the same as keratocytes in the normal corneal stroma, because corneal fibroblasts are activated in response to inflammation [24,25]. Considering that AK causes inflammation of the cornea, corneal fibroblasts rather than corneal keratocytes may be more suitable for analysis of interactions between corneal stromal cells and *Acanthamoebae*. In this study, Annexin V expression was used to detect early apoptosis and PI-positive cells were regarded as necrotic cells. Therefore, we could not determine the actual proportion of necrotic cells, because PI-positive cells resulting from apoptosis were not completely excluded. However, we at least did not overestimate the number of apoptotic cells. Further studies need to be performed to determine the exact percentage of apoptotic cells. *Acanthamoebae* express a trans-membrane protein with the characteristics of a cell surface receptor, which is called mannose-binding protein (MBP) and mediates adhesion to the surface of the cornea. Following MBP-mediated adhesion to host cells, the amoebae produce a contact-dependent metalloproteinase and several contact-independent serine proteinases [26]. Kinnear [27] reported indirect cytopathic effects of Acanthamoeba on corneal fibroblasts using insert culture dishes, suggestive of contact-independent serine proteinases. This may be because approximately 400× higher number of *Acanthamoebae* were employed in the study than our experimental setting. Considering that *Acanthamoebae* used in our experiment was enough number to kill directly activated corneal fibroblasts and *Acanthamoebae* are sparse in the corneal stroma of patients with confocal microscopic observation [28], indirect cytopathic effects of *Acanthamoebae* on corneal fibroblasts can be at least ignorable in an actual clinical setting.

In summary, we showed that *Acanthamoebae* from AK patients had a cytopathic effect on human corneal fibroblasts by direct adhesion rather than soluble mediators. This cytopathic effect on corneal fibroblasts was mainly due to apoptosis. Our findings provide some clues to the pathophysiology of corneal keratocyte loss in human AK.
